# Supplementary material for: Impact of Subclinical Haemoproteus columbae Infection on Farmed Domestic Pigeons from Central Java (Yogyakarta), Indonesia, with Special Reference to Changes in the Hemogram
Source: Pathogens. 2021 Apr 7;10(4):440. doi: 10.3390/pathogens10040440 (PMC8067996; doi:10.3390/pathogens10040440)

Supplementary Figure S1: Maximum likelihood phylogenetic trees of *Haemoproteus* spp. of the subgenus *Haemoproteus* based on the *cytb* sequences (465 characters). The species name (for the new isolates in this study with their isolate names; arrows) is followed by the MalAvi lineage tag, and DDBJ/EMBL/GenBank accession number in parentheses.

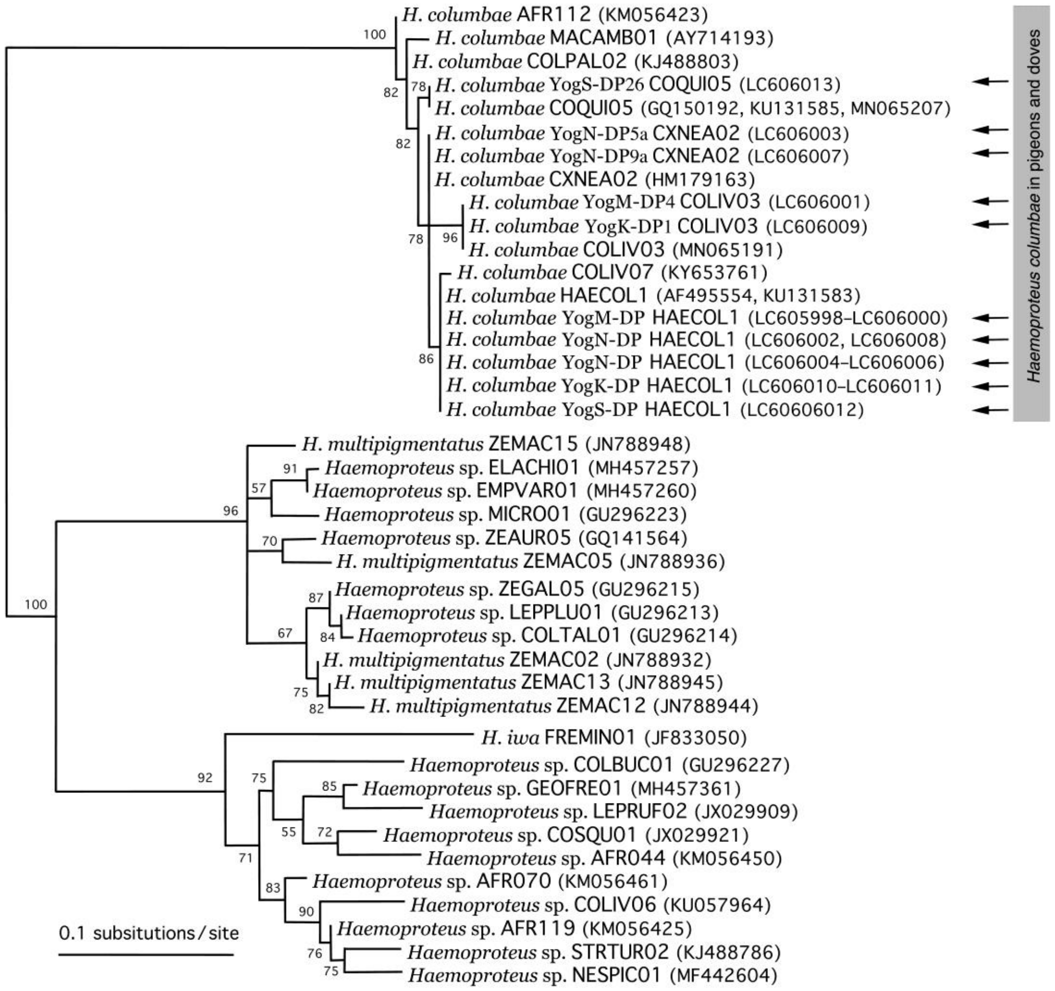

Supplement: Supplementary file 1 [file pathogens-10-00440-s001.pdf]
